# A Case Report of Intraoperative Shock Following Spinal Anesthesia: Could It Be All of the Above?

**DOI:** 10.7759/cureus.118032

**Published:** 2026-10-16

**Authors:** Ana Sousa, Rita Saraiva, Pedro Camões, Rita Borges

**Affiliations:** 1 Anesthesiology, Unidade Local de Saúde de Coimbra, Coimbra, PRT; 2 Anesthesiology, Unidade Local de Saúde da Guarda, Guarda, PRT; 3 Emergency Medicine, Instituto Nacional de Emergência Médica, Coimbra, PRT; 4 Anesthesiology, Unidade Local de Saúde da Cova da Beira, Covilhã, PRT; 5 Anesthesiology, Clinical Academic Center of Beiras, Covilhã, PRT

**Keywords:** anaphylactic shock, cardiovascular collapse, intraoperative hypotension, perioperative anaphylaxis, point-of-care echocardiography

## Abstract

Perioperative cardiovascular collapse may result from several life-threatening conditions with overlapping clinical manifestations, making the diagnosis challenging. We present a case of a 37-year-old man with morbid obesity who underwent fixation of a tibial plateau fracture. Following an uneventful ultrasound-guided femoral nerve block and spinal anesthesia, intravenous cefazolin was administered. Within minutes, the patient developed lightheadedness, nausea, loss of consciousness, jaw rigidity, and severe hypotension, refractory to fluid administration and vasopressor boluses, followed by bradycardia and respiratory distress. Point-of-care echocardiography showed preserved biventricular function and findings suggestive of marked hypovolemia, without right ventricular dysfunction. One hour after hemodynamic stabilization, the patient developed a maculopapular rash. Serial serum tryptase concentrations were markedly elevated, supporting the diagnosis of perioperative anaphylaxis. The patient recovered uneventfully and was referred to an allergy and immunology specialist. This case highlights the complexity of diagnosing sudden cardiovascular collapse during neuraxial anesthesia when several potentially life-threatening conditions are considered. Perioperative anaphylaxis should always be considered in cases of unexplained severe hypotension, even in the absence of bronchospasm or cutaneous manifestations.

## Introduction

Intraoperative hypotension is a common complication during both general and neuraxial anesthesia [1,2]. Its etiology is multifactorial, with overlapping clinical manifestations that make the differential diagnosis of cardiovascular collapse particularly challenging [1-3]. Potential etiologies include vasovagal reactions, hypovolemic shock, high or total spinal block, local anesthetic systemic toxicity (LAST), pulmonary embolism, fat embolism, and anaphylaxis [2,4].

We report the case of a patient who developed severe hypotension immediately after spinal anesthesia and cefazolin administration, highlighting the diagnostic challenges that arise when several life-threatening conditions present with similar clinical features.

## Case presentation

A 37-year-old man weighing 145 kg, with a body mass index of 44.8 kg/m² and an American Society of Anesthesiologists physical status classification of III, was scheduled for surgical fixation of a tibial plateau fracture. His medical history was notable for morbid obesity. He had no known allergies, was not taking any chronic medications, and had a previous uncomplicated inguinal hernia repair under general anesthesia.

The patient was admitted to the emergency department (ED) following a syncopal episode that resulted in a motor vehicle accident. He had no personal or family history of previous syncopal episodes. Initial evaluation revealed a right tibial plateau fracture with no evidence of cranial, thoracic, or abdominal injuries. Laboratory tests showed no significant abnormalities. Cardiology suspected a possible accessory cardiac conduction pathway (QRS slurring resembling a delta wave only in V4-V5, a PR interval of 120 ms in lead II, QRS duration <120 ms, and an rR′ pattern in V1 with ST-segment concordance rather than the discordance more commonly observed in accessory pathways), but deemed no urgent intervention necessary. Transthoracic echocardiography revealed no significant structural or functional abnormalities.

In the operating room, an ultrasound-guided femoral nerve block was performed uneventfully using 10 mL of 0.375% ropivacaine. The patient was awake, and no sedation was administered before or during the block. Subsequently, ultrasound-assisted single-shot spinal anesthesia was performed in the sitting position, using a 25-gauge, 120-mm Quincke needle at the L3-L4 interspace, and 11 mg of hypobaric levobupivacaine and 2 μg of sufentanil were administered. Immediately after intrathecal injection, intravenous cefazolin was administered for surgical antibiotic prophylaxis.

Within two minutes, the patient complained of lightheadedness and nausea, followed by rapid loss of consciousness. The episode was accompanied by jaw rigidity and severe hypotension (arterial blood pressure 54/45 mmHg), refractory to intravenous fluid administration and repeated boluses of ephedrine and phenylephrine. Bradycardia subsequently developed (heart rate 40 beats per minute) and responded to 0.5 mg of atropine. A senior anesthesiologist was immediately called for help.

The patient then developed respiratory distress with oxygen desaturation (peripheral oxygen saturation 76%), requiring tracheal intubation using videolaryngoscopy. Bilateral and symmetrical breath sounds were present without wheezing or other adventitious sounds. Persistent severe hypotension, with blood pressure becoming unmeasurable using noninvasive monitoring, prompted the initiation of a norepinephrine infusion, followed by placement of arterial and central venous catheters. To exclude cardiogenic shock, a cardiologist performed transthoracic echocardiography. It demonstrated preserved biventricular function and findings suggestive of marked hypovolemia, including ventricular “kissing” walls and a collapsed inferior vena cava, without evidence of right ventricular dysfunction or pulmonary hypertension.

Approximately one hour after the event, following hemodynamic and ventilatory stabilization, a maculopapular rash developed over the upper limbs and thorax. Hydrocortisone (200 mg) and clemastine (2 mg) were administered, fluid administration was maintained, and serum tryptase samples were collected. Cranial computed tomography (CT) and CT pulmonary angiography were performed to exclude neurological pathology and pulmonary embolism, respectively.

The patient remained hemodynamically stable in the postanesthesia care unit. Vasopressor support was gradually weaned, and he was successfully extubated. Following extubation, he was alert, neurologically intact, hemodynamically stable, and breathing comfortably with supplemental oxygen. No further cutaneous manifestations developed.

Surgery was postponed pending further investigation. A pulmonology assessment was requested because of suspected obstructive sleep apnea, while cardiology follow-up was maintained to investigate the syncopal episode that had preceded the trauma.

The markedly elevated acute serum tryptase concentration (Table 1), together with the clinical presentation, supported the diagnosis of perioperative anaphylaxis as the cause of the cardiovascular collapse. The patient was referred for an allergy and immunology evaluation to identify the causative allergen.

**Table 1:** Serial serum tryptase concentrations measured at rash onset, one hour later, and 24 hours after the event Reference value: <11.4 μ/L

Timing of blood sampling	Serum tryptase concentrations
Onset of rash	84.3 μ/L
One hour later	20.6 μ/L
24 hours later	4.1 μ/L

## Discussion

The differential diagnosis of perioperative severe hypotension is broad, particularly when multiple drugs have been administered within a short period. Differential diagnoses include high or total spinal block, LAST, hypoxia, vasovagal reaction, hypovolemic shock, pulmonary embolism, fat embolism, and anaphylaxis [2,4].

Total spinal block is characterized by sudden hypotension, bradycardia, respiratory insufficiency, loss of consciousness, and potentially apnea [5]. Given that our patient developed profound hypotension and bradycardia shortly after spinal anesthesia, this diagnosis was initially considered.

LAST was another important differential diagnosis. Although peripheral nerve blocks carry a risk of LAST, this complication is rare. Neurological manifestations, such as confusion, altered mental status, and apnea, as well as cardiovascular signs like progressive hypotension, bradycardia, and tachycardia, should raise suspicion of LAST [6,7]. In our patient, the prodromal symptoms of lightheadedness and nausea preceding cardiovascular collapse could raise suspicion of LAST [8]. In this case, the femoral nerve block was ultrasound-guided, which may reduce the risk of LAST, and aspiration before injection was negative for blood [9]. Furthermore, the relatively small dose of ropivacaine used for the femoral nerve block made this diagnosis less likely.

Thromboembolic events, including pulmonary and fat embolism, are recognized causes of perioperative complications. They may present with hypotension, desaturation, transient bronchospasm, and, in severe cases, bradycardia with increased right ventricular afterload, as shown on echocardiography by right ventricular dilation and dysfunction [6]. Thromboembolism was considered because the patient developed severe hypotension and hypoxemia. Nevertheless, transthoracic echocardiography showed preserved biventricular function without right ventricular dilation or pressure overload, and CT pulmonary angiography excluded pulmonary embolism.

Ultimately, perioperative anaphylaxis proved to be the most likely diagnosis. The incidence of perioperative anaphylaxis is approximately one per 10,000 patients, with an estimated mortality rate of 1-4% [10-13]. Antibiotics are among the most common causes of perioperative anaphylaxis, whereas amide local anesthetics and opioids are rarely implicated [9-11,14]. The initial clinical manifestations of anaphylaxis are nonspecific and may include tachycardia, hypotension, confusion, and altered mental status [6,11]. Tachycardia can evolve into bradycardia, or bradycardia can present first, particularly in severe reactions, due to a sudden decrease in peripheral resistance and reduced venous return, resulting in paradoxical bradycardia [10-12]. In such cases, atropine may be ineffective and can induce circulatory arrest [10]. Cutaneous manifestations, such as erythema and urticaria, may be absent during the initial phase of severe anaphylaxis and may appear only after hemodynamic stability is restored, because they occur only when peripheral perfusion is maintained [4,10,11]. In our patient, the rash became apparent after peripheral perfusion was restored, about one hour after hemodynamic stabilization. Bronchospasm did not precede cardiovascular instability, although respiratory manifestations may be more common in patients with obesity [10,11].

Profound vasodilation associated with anaphylaxis markedly reduces venous return and causes relative hypovolemia [6,11]. This effect was probably exacerbated by the sympathetic blockade induced by spinal anesthesia, contributing to the severity of the cardiovascular collapse. Current recommendations advocate administering prophylactic antibiotics several minutes before anesthesia induction whenever feasible, allowing earlier recognition of hypersensitivity reactions before anesthetic techniques cause hemodynamic effects [11].

Although the diagnosis of anaphylaxis was delayed, management appropriately prioritized airway protection, oxygenation, fluid resuscitation, hemodynamic support, and an early call for assistance [10]. Epinephrine remains the first-line treatment for perioperative anaphylaxis and should be administered promptly whenever anaphylaxis is suspected [10]. In this case, norepinephrine was initiated for refractory hypotension, restoring hemodynamic stability before the diagnosis became evident, and epinephrine was not administered.

This case also highlights the important role of point-of-care echocardiography in evaluating perioperative shock. The absence of ventricular dysfunction or right heart strain made cardiogenic shock and pulmonary embolism less likely, while ventricular “kissing” and a collapsed inferior vena cava suggested markedly reduced ventricular filling, guiding appropriate hemodynamic management.

Although surgery could theoretically have proceeded after stabilization, it was ultimately postponed because no operating room time was available [10,13]. However, the time-sensitive nature of the surgery precluded completion of the allergy and immunology evaluation before surgery [13].

The patient underwent surgery one week later, prior to diagnostic confirmation, due to a malfunction of the tryptase assay equipment. Because the causative allergen had not yet been identified, an alternative anesthetic technique (general anesthesia) was used, avoiding drugs administered during the previous anesthetic; alternative antibiotics (clindamycin and gentamicin) were administered for surgical prophylaxis; and fentanyl patient-controlled analgesia was initiated for postoperative pain management. The patient underwent surgery uneventfully, highlighting the challenges of managing time-sensitive procedures when the causative agent of a severe hypersensitivity reaction has not yet been identified. After the serum tryptase concentrations were determined, the patient was referred for an allergy and immunology evaluation two weeks later to identify the causative allergen.

## Conclusions

This case highlights the complexity of diagnosing sudden cardiovascular collapse during spinal anesthesia when several potentially life-threatening conditions are considered in the differential diagnosis. Perioperative anaphylaxis should always be considered in cases of unexplained severe hypotension, even in the absence of bronchospasm or cutaneous manifestations, as these signs may be delayed or absent. Early supportive management, including airway protection, oxygenation, fluid resuscitation, and hemodynamic support, is essential, while epinephrine should be administered promptly when anaphylaxis is suspected. Point-of-care echocardiography is a valuable adjunct for rapid assessment of perioperative shock, helping differentiate potential causes and guide timely therapeutic decisions.
